# Short-term and long-term effects of microplastics and organic UV-filters on the invertebrate model species *Daphnia magna*

**DOI:** 10.1007/s11356-025-36008-z

**Published:** 2025-02-01

**Authors:** Réka Svigruha, István Fodor, Zoltán Németh, Anna Farkas, Zsolt Pirger, András Ács

**Affiliations:** 1 Ecophysiological and Environmental Toxicological Research Group, HUN-REN Balaton Limnological Research Institute, 8237 Tihany, Hungary; 2 National Laboratory for Water Science and Water Security, HUN-REN Balaton Limnological Research Institute, 8237 Tihany, Hungary

**Keywords:** Microplastics, Organic UV-filters, *Daphnia magna*, Physiology, Behavior, Enzymatic biomarkers

## Abstract

There is an ongoing debate regarding the role of microplastics (MPs) in enhancing the effects of various chemical compounds, highlighting the need for more detailed analyses. In this study, neonates of the water flea (*Daphnia magna*) were exposed to polystyrene MPs (PS-MPs; 3 µm; exposure concentration, 1.25 mg/L), a mixture of seven organic UV-filters (avobenzone, ethylhexyl triazone, homosalate, iscotrizinol, octinoxate, octisalate, and octocrylene; each at a low environmental concentration of 200 ng/L), or the combination of both pollutants for 3, 7, or 21 days. Results showed that PS-MPs alone decreased the body size of daphnids, while all treatments increased heart rate by the end of the 21-day exposure. On days 3 and 21, both PS-MPs and PS-MPs + UV-filters reduced swimming speed and total distance travelled. Additionally, PS-MPs increased the time of the first egg production, but decreased the egg number in the first production, total egg number, maximum egg number, and total neonate number during the 21-day treatment. Similarly, UV-filters or the combined pollutants increased the time of the first egg production and decreased the total neonate number. All treatments increased multixenobiotic resistance activity on days 3 and 7, while only UV-filters elevated CYP450 activity on day 3. PS-MPs or combined pollutants increased GST activity during early exposure but showed no effect on day 21. CAT activity was also affected by treatments in a time-dependent manner. These findings demonstrate that chronic exposure to PS-MPs and UV-filters, applied individually or in combination at a low environmental concentration, moderately impacts development, heart rate, and swimming activity in *D. magna*, while significantly altering reproduction and key cellular functions such as membrane transport activity, metabolism, and antioxidant defense. Co-exposure did not reveal a clear pattern of synergism or antagonism, suggesting that joint toxicity risks of these xenobiotics typically emerge at concentrations higher than low environmental levels. Future studies should explore potential interactions more thoroughly and assess transgenerational effects on reproduction and cellular defense pathways.

**Supplementary Information:**

The online version contains supplementary material available at 10.1007/s11356-025-36008-z.

## Introduction

In recent years, the presence of microplastics (MPs) in aquatic ecosystems has received significant attention in ecotoxicological studies and mass media. MP pollutants are ubiquitous, reported not only in terrestrial and freshwater environments close to urban centers but also in remote areas, including the shores of uninhabited islands (Bergmann et al. 2017), snow from the Alps (Bergmann et al. 2019), Arctic snow and surface waters (Barrows et al. 2018; Bergmann et al. 2019; Materic et al. 2020), and the deep seafloor (Bergmann et al. 2017). In European rivers, the concentration of MPs was reported to range from 0.03 to 187 000 particles/m^3^ (Leslie et al. 2017; Mani et al. 2019), whereas in European lakes, it varies between 0 and 7.3 particles/m^3^ (Tanentzap et al. 2021). Highlighting, Lake Balaton, the largest shallow lake in Central Europe, contains 21.0 ± 12.5 particles/m^3^ in the size range of 50 µm–1 mm (Prikler et al. 2024).

A large body of literature has demonstrated the individual effects of MPs on algae, cnidarians, mollusks, arthropods, echinoderms, and fish (reviewed by (Albarano et al. 2024; Huang et al. 2021; Li et al. 2023; Yang et al. 2024). Moreover, it has been clearly shown that MPs can enter the human body through ingestion, inhalation, and skin contact, where they can accumulate, cross biological barriers, and pose a significant health risk (Lee et al. 2024; Li et al. 2024). In addition to their single effects, MPs exhibit a high sorption capacity for various compounds (Lara et al. 2021; Leng et al. 2023; Santana-Viera et al. 2021; Wang et al. 2021). Consequently, they can increase the toxic effects of chemical pollutants, such as polycyclic aromatic hydrocarbons or drug residues (Chen et al. 2022; Felten et al. 2020; Ghasemi and Shadi 2024; Gonzalez-Soto et al. 2019; Kim et al. 2023; Nobre et al. 2024; Wang et al. 2022; Zhang et al. 2019). However, the role of MPs in enhancing the effects of various compounds is debated, as some studies report no additive changes or suggest that MPs may mitigate the adverse effects of chemical pollutants (de Mello Souza et al. 2023; Hanslik et al. 2022; Horton et al. 2018; Ma et al. 2016; Martinez-Alvarez et al. 2024; Wang et al. 2023a; Yu et al. 2022). Based on these findings, further and more detailed analyses are clearly needed to better understand the complex effects of MPs on aquatic animals. Within the vast array of anthropogenic contaminants, the ubiquitous presence of common personal care products, such as the organic UV-filters, in recreational surface waters raises concerns on the joint ecological risks of MPs and UV-filter compounds. Given that organic UV-filtering compounds are present in the commercial sunscreen products in mixtures, they also co-occur in different environmental matrices including water and sediment (reviewed by (Kwon and Choi 2021; Mitchelmore et al. 2021)). Consequently, investigation of the interactive effects of MPs and environmentally relevant UV-filter mixtures in aquatic biota is of great significance in ecotoxicology.

To contribute to this topic, the present study was undertaken to investigate the single and combined effects of MPs with organic UV-filters compounds. For this purpose, specimens of the water flea (*Daphnia magna*) were exposed to MPs, a mixture of organic UV-filters, or the combination of the two types of pollutants for 3, 7, or 21 days. For MP exposure, polystyrene MPs (PS-MPs) with a particle size of 3 µm and a concentration of 1.25 mg/L were used, as these conditions have previously been shown to affect the physiology of *D. magna* (Svigruha et al. 2023). For UV-filter exposure, seven organic compounds commonly used in commercially available sunscreen products in Europe were selected: avobenzone (AVO), ethylhexyl triazone (EHT), homosalate (HMS), iscotrizinol (DBT), octinoxate (EHMC), octisalate (OS), and octocrylene (OC). The concentration of these compounds, except for EHT and DBT that have not yet been studied, ranges from a few ng/L to a few µg/L in surface waters (Balmer et al. 2005; Bratkovics et al. 2015; Fenni et al. 2022; Hejji et al. 2023; Nurerk et al. 2020; Rodil et al. 2009; Sanchez Rodriguez et al. 2015; Tsui et al. 2014, 2019). Based on the environmental concentrations and relevant previous acute and chronic LC_50_ data (Boyd et al. 2021, 2023), the applied exposure concentration of UV-filters was 200 ng/L (i.e., each compound was present in the mixture at a concentration of 200 ng/L). During the exposure, changes at the individual level (growth, heart rate, reproduction, swimming behavior) and cellular level (activity of enzymes participating in xenobiotic detoxification and antioxidant defense pathways) were investigated. Our findings revealed that chronic exposure of *D. magna* to environmentally relevant concentrations of PS-MPs and organic UV-filters, applied either individually or in combination, induced moderate effects across multiple biological levels. However, the potential threat of PS-MPs as vectors for organic UV-filters may be limited.

## Materials and methods

### MPs and UV-filter compounds used for the exposures

A commercially available polystyrene (PS) suspension (5% w/v, particle size of 3 µm, #42922; Merck, Hungary) was used for MP treatment. The basis for choosing PS-MP was that this polymer type is one of the most frequent MPs in the freshwaters (Koelmans et al. 2019) and is predominantly used in ecotoxicological studies (Svigruha et al. 2023).

For exposure with organic UV-filters, the following chemicals were used: AVO (CAS number: #70356–09-1), EHT (CAS number: #88122–99-0), HMS (CAS number: #118–56-9), DBT (CAS number: #154702–15-5), EHMC (CAS number: #5466–77-3), OS (CAS number: #118–60-5), and OC (CAS number: #6197–30-4). All compounds were purchased from Merck. From the UV-filter standards, 100 mg/L individual standard stock solutions were prepared by dissolving 1-mg standards in 10-mL methanol. From these stock solutions, a working solution was prepared in methanol in which the equi-concentration of the seven compounds was 5 µg/mL (i.e., each compound was at a concentration of 5 µg/mL) and stored at room temperature (22 °C) protected from light for up to 1 month. The final concentration of methanol as a carrier solvent in the experimental beakers was ≤ 0.004% v/v. Based on the results of previous publications (David et al. 2012; Dom et al. 2012; Juan-Garcia et al. 2023; Németh et al. 2024), this amount has no effect on *D. magna*.

### Experimental animals

*D. magna* specimens were obtained from our laboratory-bred culture (HUN-REN BLRI, Tihany, Hungary) initiated from cysts every third year as described in Ács et al. (2023). The animals are maintained in a controlled climate chamber (FOC 200E Connect) at 20 ± 1 °C, under a 16-h:8-h light:dark photoperiod with natural wavelength light at an intensity of 800–1000 Lux. They are cultured in 500-mL glass beakers containing 450 mL-pre-oxygenated artificial water (AFW), renewed twice a week, at a density of 2 individual/50 mL. The AFW is composed of a 1:1 v/v mixture of reverse-osmosis water and a commercial spring water (Mizse, Hungary). Water parameters, including ion composition and conductivity, are detailed in Németh et al. (2024). The daphnids are fed on *Scenedesmus obliquus* (0.5 × 10^−6^ cells/mL) three times a week. For the exposures, specimens were first randomly selected from the culture and individually placed into beakers. From these beakers, parthenogenetic neonates (< 24 h) from the third brood were randomly selected for the experiments.

### Acute and chronic exposures

The neonates were divided into four groups: (a) control, (b) MP-exposed, (c) UV-filter-exposed, and (d) MP + UV-filter-exposed. In *Experiment 1*, changes at the individual level were investigated. Neonates (*n* = 10) of the control group were individually placed into 150-mL glass beakers containing 100-mL AFW (i.e., 1 individual/100 mL) without any solvent, MP, or UV-filters. Animals in the treated groups (*n* = 10 neonates/group) were also placed individually into 150-mL glass beakers containing 100-mL AFW with PS-MP at a concentration of 1.25 mg/L (MP-exposed) or 200 ng/L equi-concentration of the seven UV-filters (UV-filter-exposed) or their mixture (MP + UV-filter-exposed). The duration of the exposure was 21 days. Each experimental group had three replicates (*n* = 30 total animals/experimental group). In *Experiment 2*, changes at the cellular level were investigated. Groups of neonates (*n* = 20) were exposed to MPs or UV-filters or their mixture at the same concentrations as described above for 3, 7, or 21 days in 500-mL media in 750-mL glass beakers. The control animals were also kept together in 500-mL media without any solvent, MP, or UV-filters for the same duration. Each experimental group had three replicates (*n* = 60 total animals/experimental group).

Based on our previous recovery data for organic UV-filter compounds (Németh et al. 2024), the artificial water in the glass beakers in both experiments was completely changed every third day and PS-MPs and/or UV-filters were re-added to maintain the nominal exposure concentrations. In both *Experiments 1* and* 2*, the specimens were always fed after the water changes. During the exposures, the incubation conditions were the same as in the case of the base culture.

### Experimental setup and physiological trait assessments

In *Experiment 1*, mortality, growth, heart rate, and reproduction were monitored during the 21-day exposure according to the description of our previous studies (Németh et al. 2024; Svigruha et al. 2021, 2023). Briefly, the growth was calculated every second day by measuring the length and width parameters (i.e., body size). The reproduction was monitored daily following the standard reproductive parameters (e.g., time to the first egg production, total neonate number). The heart rate was evaluated every third day by making 1-min video records. All investigations were carried out with a LEICA M205C stereomicroscope (BioMarker Ltd, Hungary) equipped with a LEICA DFC450 camera and evaluated with LAS software (version: 4.12). The swimming behavior was examined on days 3 and 21 by recording the swimming individuals for 1 min with a German C-mount camera (#VE10320; Loligo Systems). The records were analyzed with the EthoVision XT 17.5 software (Noldus, the Netherlands).

In *Experiment 2*, the multixenobiotic resistance (MXR) and cytochrome P450 (as ECOD) activities (in vivo) of individual specimens were investigated on the 3rd and 7th days of exposure, while the glutathione S-transferase (GST) and catalase (CAT) activities (in vitro) of pooled animals were examined on the 3rd, 7th, and 21st day of the exposure. The enzyme activity assays were performed according to the methods described in our previous studies (Ács et al. 2023, 2024; Németh et al. 2024; Svigruha et al. 2023). Briefly, for the ECOD and MXR activity measurements, ten individuals were randomly selected from each replicate of each experimental group and individually placed in the wells of 96-well-round bottom clear plates. MXR activity (i.e., ABC transporter activity) was assessed based on the calcein-AM accumulation assay described by Georgantzopoulou et al. (2016). ECOD activity was quantified as the activity of CYP450s mediating the conversion of the substrate 7-ethocycoumarin-O-deethylase to 7-hydroxycoumarin. This method was initially established by Gottardi et al. (2016) and is applicable to pooled samples of ten daphnids per replicate. Both methods were further optimized by our team to apply them for daphnids individually from the 3rd day of age onward (Ács et al. 2023, 2024). To investigate the activity of GST and CAT, ten individuals were randomly selected from each replicate of each experimental group and pooled per replicate (i.e., n = 3 pooled samples/experimental group). The samples were homogenized in phosphate buffer saline. Following centrifugation, the resulting supernatants were aliquoted according to the assay requirements set in the protocols of the assay kits applied (GST Assay Kit, #CS0410, Merck, Germany; Catalase Assay Kit, #A22180, Invitrogen, USA). Detailed methodology and calculations for all measurements are presented in the Supplementary information. All measurements were performed using a CLARIOstar^*Plus*^ (BMG LABTECH, Germany) multimode plate reader.

### Statistical analysis

Statistical analysis was performed using OriginPro 2024 software (OriginLab Corp., USA) and R v4.2.0 (R Core Team 2022). The normality of the datasets was investigated using the Shapiro–Wilk test or the Kolmogorov–Smirnov test, and the homogeneity of variances between groups was investigated using Levene’s statistics. In the case of body size and heart rate, two-way repeated-measures ANOVA was performed to study the effect of time, treatment, and time × treatment interaction. This analysis was followed by ANOVA and Tuckey’s post hoc tests or Kruskal–Wallis test with Dunn’s post hoc test to identify significant differences at the given time points (observation days). The reproduction data was analyzed with the Kruskal–Wallis test with Dunn’s post hoc test. The different enzymatic activity data were analyzed using one-way ANOVA with Tukey’s post hoc test.

## Results

### Long-term effects on growth, heart rate, and swimming behavior

**Fig. 1:**
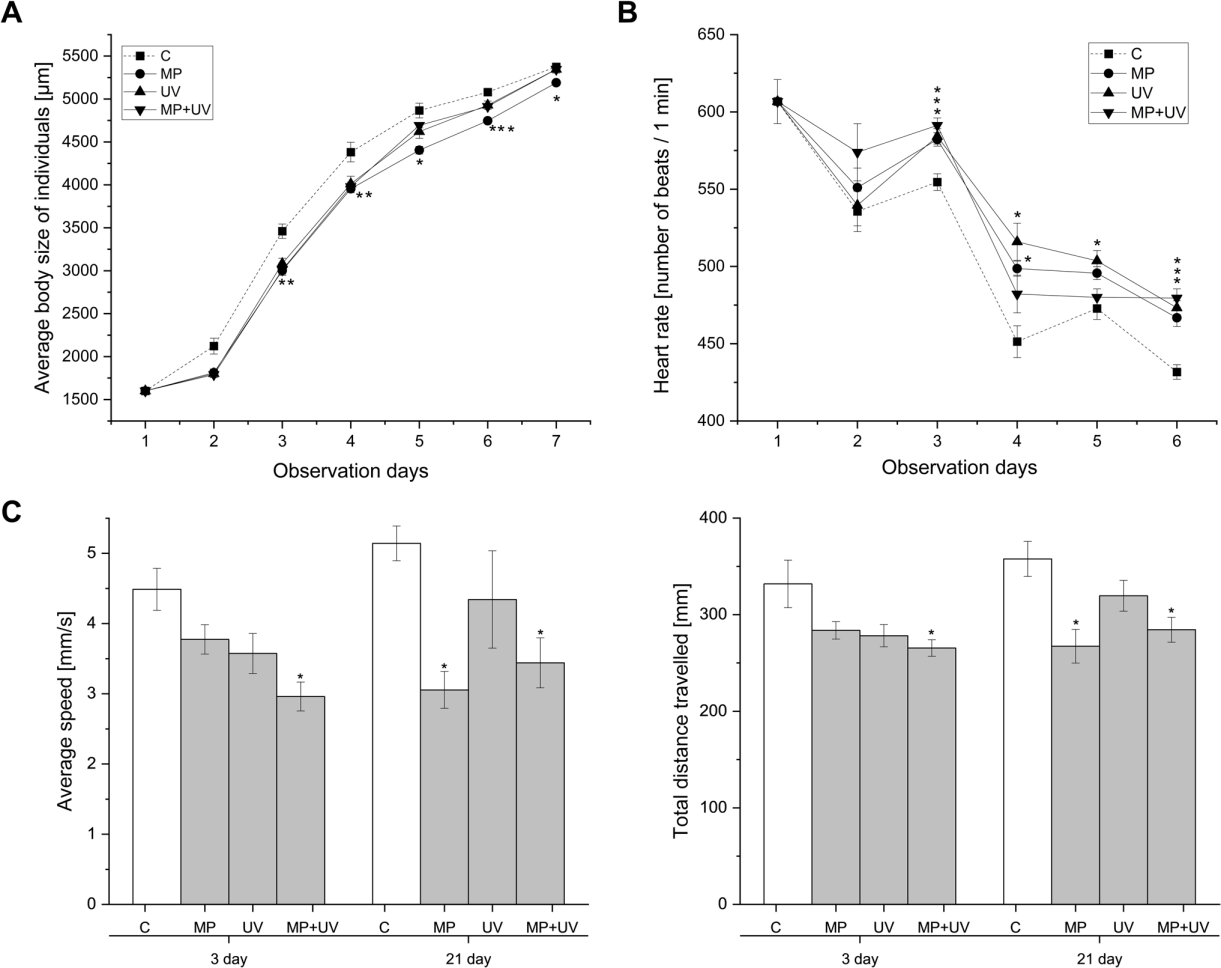
Changes in the body size (**A**), heart rate (**B**), and swimming activity (**C**) of *D. magna* specimens in the control, microplastic-treated (MP), organic UV-filter-treated (UV), and microplastic + UV-filter-treated (MP + UV) groups during the 21-day exposure. Each data point represents the mean ± SEM (*n* = 10 animals/replicate/group). Significant differences to the control group are marked by asterisks (*P < 0.05, **P < 0.01, ***P < 0.001)

The effects of exposure on growth (body size), heart rate, and swimming behavior are presented in Fig. 1. In the case of the course of growth (Fig. 1A), two-way repeated-measures ANOVA revealed significant effects of time (observation days) (*F*_6,604_ = 1555, *p* ≤ 0.001) and treatment (*F*_3,604_ = 40, *p* ≤ 0.001), but not significant with time × treatment interaction (*F*_15,604_ = 2, *p* ≤ 0.05). Further analysis with Kruskal–Wallis and Dunn’s post hoc test indicated that, compared to the control, the body size of the MP-treated group significantly decreased from the third observation day to the end of the experiment. Throughout the 7-day exposures, there were no significant differences in the body size of daphnids exposed to the MP + UV mixture or to the UV-filter mixture only.

The effect of treatment on the heart rate of daphnids during the chronic exposure is shown in Fig. 1B. Two-way repeated-measures ANOVA revealed significant effects of time (observation days) (*F*_5,477_ = 85, *p* ≤ 0.001) and treatment (*F*_3,477_ = 14, *p* ≤ 0.001), while the interaction of the two independent variables on the heart rate proved to be insignificant (*F*_15,477_ = 1, *p* > 0.05). Further analysis revealed that on the observation day 4 (*χ*^2^ = 11, *p* ≤ 0.01) and observation day 5 (*χ*^2^ = 11, *p* ≤ 0.01), the heart rate significantly increased in the UV-filter-treated group compared to the control. On the 6th observation day, the heart rate was significantly higher in all treated groups (MP: 466.8 ± 5.66, *p* ≤ 0.001; UV-filter: 473.25 ± 4.52, *p* ≤ 0.001; MP + UV-filter: 479.52 ± 5.90, *p* ≤ 0.001) than the control (431.7 ± 4.75).

The effects of exposures on the swimming behavior of daphnids were evaluated after the 3rd and 21st days of treatments (Fig. 1C). Exposure of daphnids to the MP + UV-filter mixture caused a significant decrease (by ⁓34%, *p* ≤ 0.01) in the swimming speed from the 3rd day onward, from 4.48 ± 0.29 mm/s recorded for control organisms to 2.96 ± 0.2 mm/s for exposed ones. Accordingly, by the third day of exposure, the treatment with the MP + UV-filter mixture also resulted in a significant decrease in the mean distance travelled by daphnids from 331.85 ± 2.66 mm of the control groups to 265.40 ± 8.57 mm (*p* ≤ 0.05) for the exposed groups. Neither the exposure to MPs nor to the UV-filter mixture alone caused significant alterations in the swimming speed or distance travelled by daphnids after 3 days of exposure. Following 21 days of exposure, the significantly decreased swimming speed (3.44 ± 0.36 mm/s, *p* ≤ 0.01) and distance travelled (284.42 ± 12.92 mm, *p* ≤ 0.001) persisted for daphnids treated with the MP + UV-filter mixture compared to the performance of untreated daphnids (5.14 ± 0.24 mm/s; 357.68 ± 18.18 mm, respectively). In addition, even for daphnids exposed to the MP suspension alone, significant alterations in locomotion were recorded, with a mean swimming speed of 3.05 ± 0.26 mm/s (*p* ≤ 0.01) and distance travelled of 267.39 ± 17.44 mm (*p* ≤ 0.01).

**Fig. 2:**
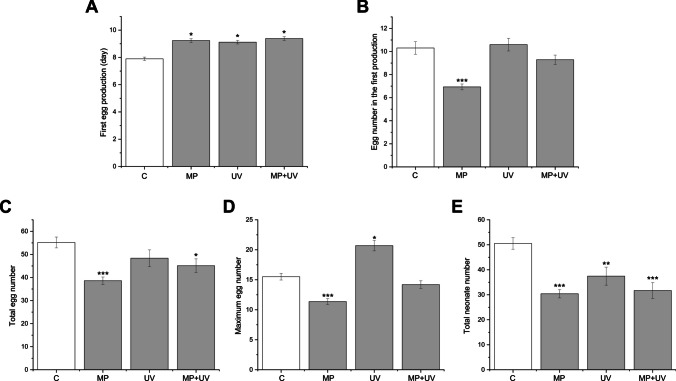
Changes in the reproduction of *D. magna* specimens in the control, microplastic-treated (MP), organic UV-filter-treated (UV), and microplastic + UV-filter-treated (MP + UV) groups during the 21-day exposure. **A** First egg production; **B** egg number in the first production; **C** total egg number; **D** maximum egg number; **E** total neonate number of individuals. Each data bar represents the mean ± SEM (*n* = 10 animals/replicate/group). Significant differences to the control group are marked by asterisks (*P < 0.05, **P < 0.01, ***P < 0.001)

Significant alterations in the reproduction performance of daphnids subjected to treatments were also recorded (Fig. 2). All treatments significantly delayed the production of the first eggs, as evidenced by the mean production times: MP, 9.23 ± 0.13 (*p* ≤ 0.001); UV-filter mixture, 9.12 ± 0.13 (*p* ≤ 0.001); MP + UV-filter mixture, 9.37 ± 0.13 (*p* ≤ 0.001); compared to controls (7.89 ± 0.12) (Fig. 2A). However, only the treatment with MPs decreased the egg number in the first production (*χ*^2^ = 38, *p* ≤ 0.001) (Fig. 2B). Compared to the control (55.17 ± 2.34), exposure to both MP (38.6 ± 1.60) and MP + UV-filters (45.10 ± 2.95) significantly decreased the total egg number (Fig. 2C). The maximum egg number laid per individual was significantly reduced in the MP-treated group (11.36 ± 0.49), while it was significantly higher in the UV-filter-treated group (20.69 ± 0.86) compared to the control (15.51 ± 0.55) (Fig. 2D). Moreover, the total neonate number was significantly reduced in all treated groups (MP, 30.40 ± 1.65, *p* ≤ 0.001; UV, 37.45 ± 3.60, *p* ≤ 0.01; MP + UV, 31.71 ± 3.15, *p* ≤ 0.001) than the control (50.55 ± 2.32) (Fig. 2E).

### Short- and long-term effects at the cellular level

**Fig. 3:**
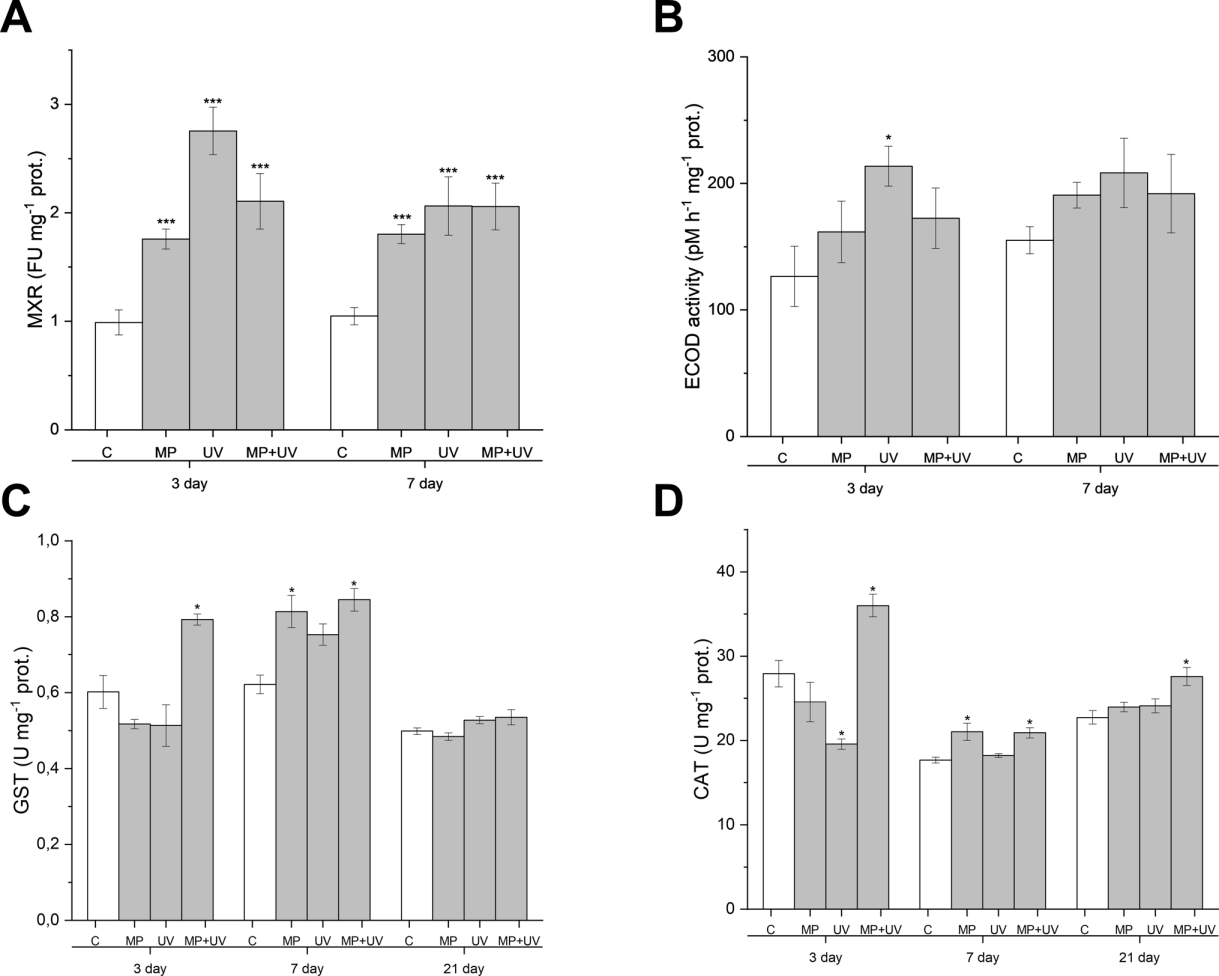
MXR (**A**), ECOD (**B**), GST (**C**), and CAT (**D**) activities of *D. magna* in the control, microplastic-treated (MP), organic UV-filter-treated (UV), and microplastic + UV-filter-treated (MP + UV) groups on the 3rd, 7th, and 21st days of treatments. Each data bar represents the mean ± SEM (A, B: *n* = 10 animals/replicate/group; C, D: *n* = 3 pooled samples/replicate/group). Significant differences to the control group are marked by asterisks (*P < 0.05, ***P < 0.001)

Changes in the activity of enzymes participating in xenobiotic detoxification and antioxidant defense pathways of *D. magna* were investigated on the 3rd, 7th, and 21st days of treatments (Fig. 3). After 3 days of exposure, the MXR activity was inhibited significantly in all treated groups (MP, 23.96 ± 0.56 FU/mg protein, *p* ≤ 0.001; UV, 24.11 ± 0.83 FU/mg protein, *p* ≤ 0.001; MP + UV, 27.59 ± 1.06 FU/mg protein, *p* ≤ 0.001) compared to the control (22.73 ± 0.80 FU/mg protein) (Fig. 3A). Conversely, Phase I xenobiotic metabolizing enzyme ECOD activity was significantly increased in the UV-treated group (213.61 ± 15.82 pM/h/mg protein, *p* ≤ 0.05; control, 126.54 ± 23.91 pM/h/mg protein) (Fig. 3B), while Phase II GST enzyme activity was significantly elevated in daphnids subjected to the MP + UV-filter treatments (0.54 ± 0.02 U/mg protein, *p* ≤ 0.05; control, 0.50 ± 0.01 U/mg protein) (Fig. 3C). The activity of the antioxidant enzyme CAT was significantly inhibited following the exposure to the UV-filter mixtures (24.12 ± 0.83 U/mg protein, *p* ≤ 0.05), while it increased upon the treatment with MP + UV-filters (27.59 ± 1.06 U/mg protein, *p* ≤ 0.05) compared to control (22.73 ± 0.80 U/mg protein) (Fig. 3D).

After the first week of exposure, the MXR activity was still significantly inhibited in all treated groups (MP, 1.80 ± 0.09 FU/mg protein, *p* ≤ 0.001; UV, 2.06 ± 0.27 FU/mg protein, *p* ≤ 0.001; MP + UV, 2.06 ± 0.22 FU/mg protein, *p* ≤ 0.001) as compared to the control (1.048 ± 0.08 FU/mg protein) (Fig. 3A). A minor increase in ECOD activity was observable in all daphnids subjected to exposures, without any statistically significant difference as compared to baseline level characteristics for the control group (Fig. 3B). In the meantime, significant increases in both the GST (*F*_3,34_ = 9, *p* ≤ 0.01) (Fig. 3C) and CAT (*F*_3,81_ = 7, *p* ≤ 0.001) (Fig. 3D) activities were evidenced in daphnids subjected to MP- and MP + UV treatments, respectively.

After 3 weeks of exposure, there was no significant difference in GST activity between the experimental groups (Fig. 3C). In contrast, CAT activity was significantly increased in the case of the MP + UV-exposed group (35.00 ± 1.34 U/mg protein, *p* ≤ 0.05) compared to control (27.92 ± 1.56 U/mg protein) (Fig. 3D).

## Discussion

Several prior studies have investigated the single effects of MPs (reviewed by Samadi et al. 2022; Yin et al. 2023) or UV-filter compounds (reviewed by Chatzigianni et al. 2022) on *D. magna*, demonstrating a range of adverse effects from behavioral to molecular levels. However, to our knowledge, only one study investigated the combined effects of these two types of pollutants (Kim et al. 2023). Furthermore, in that study, only AVO as a single compound was applied for the exposure. Clearly, there is a lack of information regarding the potential combined effects of MPs and different organic UV-filtering compounds. It is noteworthy that the individual and combined effects of AVO, HMS, EHMC, OS, and OC have previously been investigated in *D. magna* (Boyd et al. 2021, 2023; de Paula et al. 2022; Gackowska et al. 2018; Lambert et al. 2021; Marcin and Aleksander 2023; Park et al. 2017), but no study has addressed EHT and DBT to date.

Earlier studies reported various lethality thresholds for PS-MP upon chronic exposures of daphnids (e.g., 2 mg/L for 0.7 µm or 5 mg/L for 6 µm PS-MPs) (De Felice et al. 2019; Jeong et al. 2022; Liu et al. 2022a, 2022b; Trotter et al. 2021; Zhu et al. 2022). In accordance with our previous findings (Svigruha et al. 2023), this study evidenced that PS-MPs alone at 1.25 mg/L concentration did not cause significant mortality during the 21-day exposure (Supplementary Table 1). Chronic LC_50_ data for AVO (42 µg/L), OCTO (5.35 µg/L), and oxybenzone (53.5 µg/L), as well as acute LC_50_ data for their 1:1:1 mixture (0.99 mg/L), were previously determined in the case of *D. magna* (Boyd et al. 2021, 2023). Accordingly, in the present study, the applied UV-filter mixture at 1.4 µg/L in total was not lethal for *D. magna* during long-term exposure. The animals were also able to tolerate 1.25 mg/L MPs and the summed concentration of 1.4 µg/L of the selected UV-filters (200 ng/L per component).

Exposure to PS-MPs was also proved to affect the body size of *D. magna*. Overall, consistent with our previous findings (Svigruha et al. 2023) and other relevant studies (Leong et al. 2001; Liu et al. 2022b; Trotter et al. 2021; Zhu et al. 2022), long-term exposure to PS-MPs led to a progressive and significant decrease in the body size of the specimens. Trotter et al. (2021) evidenced that long-term exposure of *D. magna* to polystyrene microplastics (PS-MPs) elicited a significant downregulation of various digestive enzymes, which further decreased the nutrient supply of organisms finally leading to reductions in the size of organisms by maturation. Similarly, Zhu et al. (2022) reported that exposure of *D. pulex* to PS-MPs altered the nutrient provision of organisms by inhibiting the lipid and energy metabolism; meanwhile, the fecundity was maintained at the cost of self-sustenance. The effect of UV-filters, especially in mixtures, on the body size of *D. magna* is largely underexamined. Single UV-filter compounds, including OCTO and OCTI, were demonstrated to alter the length, or the perimeter of *D. magna* during chronic exposures (Lambert et al. 2021; Németh et al. 2024). Specifically, the body size of the daphnids was 13% larger than that of the control following a 21-day exposure to OCTO at 200 ng/L (Németh et al. 2024), and the body length of daphnids was also significantly increased upon a 21-day treatment of F1 generation daphnids with OCTI at 75 µg/L (Lambert et al. 2021). However, in the present study, neither the mixture of organic UV-filters nor the combination of PS-MPs and UV-filters affected the body size of the animals. Exposure duration-dependent transient alterations in the development of daphnids primarily result from either the disruption of food intake but also due to an energy reallocation needed to maintain for example appropriate antioxidant capacity under oxidative stress conditions (Boyd et al. 2023; Zhu et al. 2022). In general, the activation of biotransformation processes and cellular repair upon toxic pressure imply increased energy requirements consumed at the cost of essential life-history traits as growth to which juvenile organisms are more susceptible. As organisms subjected to long-term exposure to low levels of contaminants are capable of adapting to such stressful conditions, the physiological alterations developing in the initial phase of toxic stress may recover in time. Nevertheless, the real risks of environmental exposure concentrations of PS-MPs and UV-filter mixtures on the development of daphnids may be assessed via multigenerational investigations (Boyd et al. 2023).

Evidence exists for both PS-MPs and various organic UV-filters to cause cardiotoxicity in various test models. Development of cardiotoxicity post exposure to polystyrene microplastics was reported in chicken, initiated via ROS-driven NF-κB-NLRP3-GSDMD and AMPK-PGC-1α axes (Zhang et al. 2022). An in vitro study demonstrated that oxidative stress elicited by exposure to PS-MPs further promoted increases in intraventricular septal thickness and myocardial hypertrophy in human pluripotent stem cells (Zhou et al. 2023). Furthermore, exposure of zebrafish larvae to the organic UV-filter ethylhexyl salicylate altered cardiovascular development by disrupting the lipid metabolism in fish (Zhao et al. 2023). Our results demonstrated that PS-MPs significantly increased the heart rate at the end of the chronic exposure. In our previous study, chronic exposure to 200 ng/L AVO, OCTO, or OCTI significantly increased the heart rate of daphnids (Németh et al. 2024). Similarly, in the present study, the heart rate was significantly increased due to the mixture of organic UV-filters. Moreover, PS-MPs and the combination of the two pollution types also enhanced the heart rate significantly. Previous studies have clearly demonstrated that PS-MPs can affect the swimming behavior of *D. magna*, although the results are contradictory. An earlier study demonstrated a decreased swimming velocity of daphnids following an acute exposure to 16 µL/L PS-MPs in the range of 8.65 µm and 23.4 µm (Magester et al. 2021). After chronic (21-day) exposure with 5 µm PS-MPs at 2.5 mg/L concentration, the swimming velocity of Daphnia individuals was significantly increased (Liu et al. 2022a). In contrast, PS-MPs (0.7 and 3 µm) at three concentrations (0, 2, and 6 mg/L) did not affect the swimming of *D. magna* (Wang et al. 2023b). In the present study, long-term (21 days) but not short-term (7 days) exposure with PS-MPs decreased the swimming speed and, accordingly, the total distance travelled of the animals. Exposure to single UV-filter compounds (100 µg/L benzophenone-3 or 200 ng/L AVO or 200 ng/L OCTI) was also shown to inhibit the swimming activity of daphnids (Németh et al. 2024; Yang et al. 2021). Interestingly, the mixture of the chemicals did not have any effect on this endpoint. However, the combination of PS-MPs and UV-filters also decreased both swimming speed and distance travelled both in the beginning and at the end of the 21-day treatment. Seemingly, the combination of the two pollution types had an additive acute effect.

Previous studies have reported inconsistent findings on the long-term effects of MPs on the reproduction of *D. magna* (summarized in (Svigruha et al. 2023). In accordance with the findings of some previous studies (De Felice et al. 2019; Liu et al. 2022c, 2022b), chronic exposure with PS-MPs delayed the first egg production. Moreover, similar to previous studies (Jeong et al. 2022; Liu et al. 2022c, 2022b; Svigruha et al. 2023; Trotter et al. 2021), the PS-MP treatment decreased the reproductive output of daphnids. The effects of organic UV-filters on the reproductive performance of *D. magna* have also been investigated, demonstrating significant changes in both egg and neonate numbers depending on the compound and concentration (Boyd et al. 2021, 2023; de Paula et al. 2022; Németh et al. 2024). Chronic exposure to AVO (20 µg/L) significantly increased the number of neonates produced per individual, but OCTO (0.5 µg/L) had no effect on this parameter (Boyd et al. 2021). Higher concentrations of AVO (25 µg/L) or OCTO (3 µg/L) caused some multigenerational effects manifested only in transient and minor decreases in the total reproductive effort (Boyd et al. 2023). Our previous study showed that a 21-day exposure to OCTO at 200 ng/L concentration significantly increased the total egg number and total neonate number produced per individual (Németh et al. 2024). Moreover, the maximum egg number was also increased significantly after a chronic exposure to AVO, OCTO, and OCTI as single compounds (200 ng/L) (Németh et al. 2024). The present study evidenced that exposure to a UV-filter mixture alone or in combination with PS-MPs significantly delayed the reproduction, reduced the total number of eggs, and decreased the survival of neonates. These effects align with previous findings (Boyd et al. 2023; Zhu et al. 2022), suggesting energy reallocation from reproductive processes to xenobiotic defense mechanisms (biotransformation, cellular repair) to ensure organism survival (Sokolova et al. 2012). This phenomenon is particularly true when organisms are affected by low-stress conditions over a limited duration.

Exposure of aquatic organisms to environmentally relevant concentrations of both PS-MPs and UV-filters has been shown to trigger various responses in basic cellular defense pathways. Recent studies indicate that all three primary detoxification mechanisms—Phase I, II, and III—may be activated or dysregulated in organisms exposed to these xenobiotics (Kazmi et al. 2024; Martinez-Guitarte 2018; Rafeletou et al. 2024). In addition, a range of immune responses, altered metabolism, neurotoxicity, apoptosis, DNA damage, and oxidative stress development were also evidenced (as reviewed by (Kazmi et al. 2024; Rafeletou et al. 2024)). In this study, we specifically focused on mapping the responsiveness over time of the Phase I and II detoxification pathways and membrane transporter activity to act against the chronic toxic pressure exerted by the two types of contaminants either individually or in a mixture. Significant alterations in the physiological traits investigated were recorded in the developmental phase of daphnids, with the highest damages recorded in 3-day-old younglings. Both PS-MPs and the UV-filter mixture individually caused significant inhibition of MXR activity in daphnids by the third day of exposure and led to a slight increase in ECOD activity, which was statistically significant only for the UV-filter mixture. The functional inhibition of ABC transporter proteins by a toxicant results not only in its own accumulation but also in the cellular sequestration of other xenobiotics present in the ambience. The continuous accumulation of toxicants in cells may further promote a range of physiological alterations (development, growth, reproduction) as well as metabolic and cellular damages that may risk even the survival of organisms (Lee et al. 2023; Prata et al. 2018; Wang et al. 2020). Speculatively, elevated ECOD activity may have occurred as the result of suppressed MXR activity. Moreover, biotransformation of xenobiotics by CYP450 isoenzymes may also lead to enhanced intracellular ROS overproduction, which in turn may imbalance the antioxidant functions in cells, finally invoking oxidative stress (Salvo et al. 2012; Wang et al. 2016). Inhibition of ABC transporter activities upon 48-h exposure to PS-MPs (2 µm, 1 mg/L) was reported in daphnids (Lee et al. 2023) or in *Mytilus galloprovincialis* larvae and adults exposed for 2 and 4 days respectively to 3 µm PS-MPs at densities of 50 and 500 particles/mL (Franzellitti et al. 2019).

Studies investigating the potential contribution of the ATB-binding cassette transporters in the cellular defense against the toxicity of UV-filters are seldom. Previous studies reported differential potency for various organic UV-filters to inhibit MXR activity in *Tetrahymena thermophila* (Gao et al. 2016) and in zebrafish (Prakash et al. 2022). Our results showed that both individual and combined exposures to PS-MPs and UV-filter mixtures caused similar inhibition of MXR activity in daphnids by the 3rd and 7th days, suggesting that both compound classes have a comparable potential to inhibit ABCB1 protein activity. The comparable MXR inhibition observed in both combined and single exposures to PS-MPs and UV-filters suggests that environmental concentrations of these xenobiotics do not exert even an additive inhibitory effect on the MXR-mediated cellular efflux in daphnids. We hypothesize that UV-filters entering the cells were mainly adsorbed on the surface of microplastic particles and did not evolve their individual toxic action. Our presumption is based on evidence that a vast array of xenobiotics can bind to the surface of particulate microplastics; thus, microplastics act as carriers or mediators of other contaminants enhancing or alleviating their toxicity in aquatic organisms (Lee et al. 2023; Prata et al. 2018; Wang et al. 2020).

The contribution of Phase I metabolism in defending the toxicity of PS-MPs and UV-filters in aquatic organisms was also reported in a few studies. Modulation of Phase I detoxification was evidenced in fish and crustaceans exposed to environmentally relevant concentrations of PS-MPs of diverse particle sizes (Daniel et al. 2024; Ding et al. 2018). Exposure dose- and time-dependent variations in EROD and BFCOD activities were detected in the liver of *Oncorhynchus niloticus*, with an initial decrease up to the 6th day of exposure then, followed by a rapid increase in activities of both isozymes within the 6–14 days of exposure with 0.1 µm PS-MPs within 1–100 µg/L dose range (Ding et al. 2018). Similarly, increased cytochrome P450 enzyme activities were detected in *D. magna* upon exposure to polyethylene MPs with particle sizes < 100 µm at densities of 0.5 and 5 mg/L, while acute exposure to polypropylene MPs of the same particle sizes and densities did not affect the baseline CYP1A1 and CYP3A4 in daphnids (Daniel et al. 2024). Regardless of exposure duration, our study found no statistically significant variation in ECOD activity in daphnids treated with 3 µm PS-MPs at a dose of 1.25 mg/L, suggesting that the CYP2 isozymes, which are most abundant in daphnids, are relatively insensitive to this level of PS-MP exposure. In contrast, exposure to the UV-filter mixture caused a moderate but statistically significant increase in ECOD activity in juvenile daphnids by the 3rd day, which returned to baseline levels by the 7th day. The significant increase in ECOD activity of younger daphnids may be related to the earlier observation that CYP 450 enzyme activity in 3-day-old daphnids is more pronounced than in their later developmental phase (Ács et al. 2024; Melo de Almeida et al. 2022). Co-exposure of daphnids to the PS-MPs and UV-filter mixture resulted in a mild, statistically insignificant increase in ECOD activity at both developmental stages, suggesting that MP particles adsorbed the UV-filters to their surface and acted as a sink of these compounds. This fact may further suggest that the bioaccumulation and toxicity of the UV-filters were alleviated by the presence of MPs in the exposure media.

Exposure studies performed on fish, bivalve, and crustacean test models demonstrated for both PS-MPs and organic UV-filters to cause various alterations patterns in the Phase II metabolism and the antioxidant defense pathways in these organisms (as reviewed by Carve et al. 2021; Kazmi et al. 2024). In crustaceans, cellular alterations elicited by exposures to PS-MPs are highly dependent on the shape, size, and applied exposure concentrations (Kazmi et al. 2024). While a number of studies reported insignificant alterations in the glutathione conjugation pathways of daphnids chronically exposed to PS-MPs (De Felice et al. 2022; Zhang et al. 2020), significant inhibition in GST activity was observed upon acute exposure (Fadare et al. 2019; Li et al. 2020). The differences observed in the modulation of GST activity in daphnids to PS-MPs as a function of exposure duration are presumed to result from the higher sensitivity of younger individuals to these types of MPs (Daniel et al. 2024). Moreover, a previous study highlighted even moderate activation of GST activity in daphnids acutely exposed to PS-MPs (Zhang et al. 2019), a phenomenon suggested to be caused by leachable chemical constituents commonly present in microplastics (Daniel et al. 2024). Studies investigating cellular alterations in aquatic organisms exposed to organic UV-filters reported compound-specific as well as dose- and exposure time-dependent specific patterns. Post accumulation in cells, organic UV-filters, as most lipophilic xenobiotics, may undergo biotransformation via cellular metabolism resulting in more reactive intermediates and overproduction of reactive oxygen species (as reviewed by Carve et al. 2021). These metabolites may further initiate the activation of Phase II metabolism and of the antioxidant defense pathways as reported in daphnids subjected to chronic exposure to avobenzone, benzophenone, and octocrylene (0.2–4.4 µg/L) (de Paula et al. 2022), in the bivalve *Mytilus edulis* sub-chronically exposed to ensulizole and octocrylene (10–100 µg/L) (Falfushynska et al. 2021), or in zebrafish larvae acutely exposed to 4-methylbenzylidene camphor (5 µg/L) (Prakash et al. 2022). Our results evidenced a moderate increase in GST activities specifically in premature daphnids following the 7th day of exposure for both contaminant classes, while the combined exposure suggested additive effects only in 3-day-old daphnids. These results support previous findings indicating higher susceptibility to these contaminants for juvenile stages and suggest that daphnids are able to adapt to currently existing environmental contamination loads of PS-MPs and UV-filters in natural surface waters.

Reactive oxygen species formation appears to be a general toxicity feature of microplastics in aquatic organisms (including crustaceans), primarily leading to disturbances of the antioxidant defense mechanisms (De Felice et al. 2022; Liu et al. 2020; Zhu et al. 2022). The resultant oxidative stress state was proved to highly depend on both the size and concentration of PS-MPs. Zhu et al. (2022) reported induction of the antioxidant defense in *D. pulex* exposed to 500-nm PS-MPs in concentrations below 2 mg/L and depressed antioxidant functions above this threshold, while even low-doses (< 0.5 mg/L) of 75-nm PS-MPs caused significant inhibition of both primary antioxidants the superoxide dismutase (SOD) and CAT activities in *D. pulex* (Liu et al. 2020). Both the aforementioned observations were recorded following acute exposures of daphnids to PS-MPs and are in apparent contradiction with the results obtained by De Felice et al. (2022) who reported unaltered oxidative state in *D. magna* chronically exposed to PS nanoplastics (50 nm; 50, 500 mg/L) as revealed by insignificant variations in SOD and CAT enzymatic activities. In fact, activation of the antioxidant system in crustaceans during short-term exposure to environmentally relevant concentrations of PS-MPs followed by a return to baseline activities upon chronic exposure exemplifies the apparent capability of these organisms to adapt to such stressful conditions. In our study, activation of the antioxidant system in daphnids exposed to 1.25 mg/L microplastics was evidenced by the significant increase in CAT activity by the 7th day of exposure, which then dropped to baseline level by the 21st day of treatment.

As mentioned earlier, organic UV-filters accumulated in aquatic organisms may undergo biotransformation to variable extents leading to the generation of more reactive intermediates and ROS overproduction, which initiate the activation of antioxidant pathways in cells (de Paula et al. 2022; Watanabe et al. 2015; Yang et al. 2021). The responsiveness of the antioxidant system proved to be highly variable as a function of the molecular structure of UV-filters, as well as of dose and exposure duration. Acute (24 h) exposure of daphnids to 100 µg/L avobenzone initiated a significant increase in CAT and SOD activities suggesting an active defense initiation against the free radicals formed (Yang et al. 2021). Increased CAT activity was recorded also in daphnids exposed for 21 days to environmental concentrations of avobenzone, benzophenone-3, and octocrylene (0.17–4.4 µg/L), which indicates that the antioxidant system of daphnids may cope with such relatively low toxic stress even for longer duration. Our results contrast with these previous findings, as exposure to a UV-filter mixture with a total concentration of 1.4 µg/L significantly inhibited CAT activity in juvenile daphnids after 3 days. By the 7th day, CAT activity returned to baseline and remained at this level through the 21st day. The initial inhibition in CAT activity may rely on the higher susceptibility of younger individuals to the toxic pressure of the UV-filter mixture tested, while further baseline activity suggests an unaltered oxidative state in mature individuals. Long-term exposure to environmental concentrations of various organic UV-filters did not affect the activities of SOD or CAT activities in trout (Grabicova et al. 2013), or in the midge larvae *Chironomus riparius* (Campos et al. 2017), demonstrating again the potential for aquatic organisms to cope with the toxic pressure of these contaminants even for a longer duration. In our study, the simultaneous exposure of daphnids to PS-MPs and the UV-filter mixture caused significantly increased CAT activity in organisms throughout the 21-day exposure with the most relevant increase as compared to control by the 3rd day of exposure. These results suggest a synergistic toxicity feature of the two contaminant classes to daphnids, but further research with a focus on the mode of toxic action of these toxicants is needed to fully support this first observation.

## Conclusions

The results of this study demonstrated that chronic exposure of *D. magna* to environmentally relevant concentrations of PS-MPs and UV-filters, applied either individually or in combination, moderately affected physiological parameters (e.g., heart rate, swimming activity) of daphnids at various time points in their life cycle. Notably, the treatments had a significant impact on the reproduction performance manifested as delayed egg production, reduced maximum egg production per reproductive cycle, and a lower number of neonates. Both individual and combined treatments elicited significant alterations in basic cellular functions such as membrane transport activity, metabolism, and antioxidant defense with more pronounced effects in juvenile organisms. Juveniles are generally more susceptible to different compounds in the water due to several physiological and biological characteristics. Speculatively, their higher metabolic rate (leading to increased uptake of water and associated physical or chemical compounds per unit of body weight) and weaker immune responses (making them more vulnerable to oxidative stress and cellular damage) contribute to the pronounced effects observed. The persistent, significant inhibition of membrane transport activities in daphnids across all treatments suggests a high risk for crustacean populations continuously exposed to these contaminants in the environment. Impaired efflux activity may facilitate the accumulation of xenobiotics and other metabolites within cells, leading to increased chemical and energetic stress, reduced resilience to environmental challenges, and decreased fitness. In this study, co-exposure of daphnids to environmental concentrations of PS-MPs and UV-filters did not clearly demonstrate a specific interactive pattern of either synergism or antagonism of these contaminants, which further supports the notion that joint toxicity risks of xenobiotics evolve in general at doses exceeding environmental concentrations. However, further, more detailed studies involving dose–response investigations are required to confirm this assumption. Nevertheless, the data obtained clearly indicate an energy allocation from reproduction to cellular processes. Since the applied concentration of organic UV-filters represents a low environmental level, even more severe effects on ecosystems could be expected. Future studies should aim to (1) investigate potential interactions in greater detail (i.e., dose–response experiments) and (2) assess transgenerational effects on reproduction and key cellular functions (e.g., xenobiotic detoxification) to reveal a more precise environmental impact of the joint presence of MPs and organic UV-filters.

## Supplementary Information

Below is the link to the electronic supplementary material.

Supplementary file1 (DOCX 38 KB)

## Data Availability

Data will be made available on request.
