# Supplementary material for: Short-term and long-term effects of microplastics and organic UV-filters on the invertebrate model species Daphnia magna
Source: Environ Sci Pollut Res Int. 2025 Feb 1;32(8):4841–55. doi: 10.1007/s11356-025-36008-z (PMC11850500; doi:10.1007/s11356-025-36008-z)
Supplement: Supplementary file 1 — Supplementary file1 (DOCX 38 KB) [file 11356_2025_36008_MOESM1_ESM.docx]

**Supplementary information**

**Short-term and long-term effects of microplastics and organic UV-filters on the invertebrate model species *Daphnia magna***

^1,2^Réka Svigruha, ^1,2^István Fodor, ^1,2^Zoltán Németh, ^1,2^Anna Farkas, ^1,2^Zsolt Pirger, ^1,2^András Ács

^1^Ecophysiological and Environmental Toxicological Research Group, HUN-REN Balaton Limnological Research Institute, H-8237 Tihany, Hungary

^2^National Laboratory for Water Science and Water Security, HUN-REN Balaton Limnological Research Institute, H-8237 Tihany, Hungary

*Corresponding author; E-mail: [svigruha.reka@blki.hun-ren.hu](mailto:svigruha.reka@blki.hun-ren.hu) (R Svigruha)

**Detailed methodology and calculations for the enzymatic activity measurements**

*Sample preparation*

To investigate the MXR and ECOD activities, living daphnids of each replicate of each experimental group were individually placed in the wells of bottom clear plates. To examine the GST and CAT activities, the animals of each replicate of each experimental group were pooled (resulted in 3 pooled samples/experimental group). The samples were homogenized in 500 μL phosphate buffer saline (0.5 M; pH = 7.4) with a TissueLyser LT device (QIAGEN, Germany) at 50 Hz for 3 min. Following centrifugation (10,000g for 15 min at 4 °C), the resulted supernatants were aliquoted according to the assay requirements set in the protocols.

*MXR activity measurement*

The MXR activity was evaluated based on the accumulation of the probe calcein-AM following in principle the method described by (Georgantzopoulou et al. 2016), which was also used in our previous study (Acs et al. 2023). After the treatments, living daphnids were individually placed in the wells of 6-well-round bottom clear plates containing 10 mL of the initial exposure media spiked with calcein-AM at a final 0.5 μM concentration and incubated in complete darkness for 2 h. After the incubation, daphnids were washed of excess dye with aerated artificial and transferred individually to wells of a 96-well round bottom clear plate. Following complete water removal, the daphnids were frozen overnight at -20 °C. Next, the animals were mechanically disrupted in 15 μL of ice-cold phosphate buffer saline (0.1 M PBS), using 5 mm stainless steel beads (1 bead/well, Qiagen GmBH, Hilden, Germany) previously cooled to -20 °C shaking the plate at 500 rpm and 4 °C for 10 min in a light protected thermostatic shaker (Thermo-Shaker TS-100C, Biosan SIA, Ratsupites, Latvia). Next, an additional 135 μL of cold 0.1 M PBS was added to the wells. The tissue homogenate and buffer were mixed gently and finally the resultant homogenates were transferred to 0.2 mL 8 Tube PCR strips (ThermoFisher Scientific, USA). Next the homogenates were centrifuged at 10,000 ×g, 4 °C for 5 min using a mySPIN 12 mini centrifuge (ThermoFisher Scientific, USA). One hundred microliters of the supernatant was used for fluorescence measurements performed at 485/14 nm excitation and 535/25 nm emission wavelengths.

*ECOD activity measurement*

The *in vivo* EROD activity assay originally developed for fish embryos (Gaaied et al. 2019; Le Bihanic et al. 2013; Schiano Di Lombo et al. 2021) was further optimized for daphnids by our research group (Acs et al. 2023). The method relies on assessing the conversion of 7-ethoxycoumarin-O-deethylase (7-ER) to 7-hydroxycoumarin (7-HCm) by Cytochrome P450 enzymes in daphnids. Specimens of *D. magna* cleaned from exposure media were individually placed in the wells of a 96-well-round bottom clear plate (Greiner Bio-One, Hungary) and preincubated in 200 µL 0.5 µM 7-ER for 1 hour at room temperature in complete darkness in order to avoid algal growth (first incubation). Next, the substrate media was completely renewed and the incubation continued for further 3 hours in complete darkness (second incubation). After both incubations, 100 µL of dye solution was sampled from each well, transferred to the wells of a 96-well black microplate (Opti Plate-96F, PerkinElmer Inc., USA), and the fluorescence was measured at 355/40 nm excitation and 460/25 nm emission wavelengths. The ECOD activity was estimated as the change in fluorescence of the incubation media within the 3 hours timeframe (both corrected to the background fluorescence of blank wells) and further quantified against a calibration curve of 7-HCm prepared within the 1 – 50 pM 7-HCm L^-1^ concentration range. Relative ECOD activity data were normalized to incubation time (minutes).

*GST activity measurement*

The GST activity was measured using a commercially available kit (GST Assay Kit, #CS0410, Merck, Germany). The assay principle relies on monitoring the conjugation of the reduced L-glutathione (GSH) by 1-chloro-2,4-dinitrobenzene (CDNB). The assay was performed in principle following the protocol instructions but modifying the sample and assay volumes to reach better performance. In this case, 20 µL of sample supernatant were assayed in a final 100 µL reaction volume, while maintaining the concentration of GSH and CDNB reagents at the prescribed levels (20 mM GSH mL^-1^; 10 mM CDNB mL^-1^). The assay was performed with two technical replicates per each sample, blank, and standard. The change in absorbance of the samples was monitored spectrophotometrically at 340 nm every minute over a period of 10 min. GST specific activity was calculated according to the following equation:

$$GST activity= \frac{\Delta A_{340}{min}^{-1}*reaction volume (mL)}{0.0096 {\mu mol}^{-1}* {cm}^{-1} *1000mml* 0.2893 cm*sample volume (mL)}$$

where:

ΔA_340_/min = the change in absorbance per minute of the reaction mixture

0.0096 µmol^-1^cm^-1^ in the extinction coefficient of the glutathione-DNB adduct

0.2893 cm is the light path of the 0.1 mL reaction volume in a 96 well plate.

*CAT activity measurement*

The CAT activity was measured using a commercially available kit (Catalase Assay Kit, #A22180, Invitrogen, USA). The principle of the assay relies on determining the decrease in H_2_O_2_ concentration following an incubation of the samples with a standard H_2_O_2_ standard solution due to its disintegration to water and oxygen (O_2_) by the catalase enzymes present in samples. In the presence of horseradish peroxidase, Amplex Red reacts with the remaining H_2_O_2_ in a 1:1 stoichiometry to produce the highly fluorescent resorufin. The assay was performed on 25 µL sample supernatant in 100 µL final reaction volume strictly following the provided assay protocol. Two technical replicates were applied for each sample, blank, and standard. The produced resorufin was quantified fluorometrically using excitation at 530 ± 25 nm and fluorescence detection at 590 ± 20 nm.

Catalase activities were calculated by means of the equation obtained from the linear regression of a calibration curve of catalase standard according to the following equation:

$$CAT\left( U/{mL} \right)=\left[ \left( \frac{\Delta FLU-y-intercept}{slope} \right)*\frac{0.1 mL}{0.01 mL} \right]$$

where:

*ΔFLU* = change in fluorescence reported as the measured fluorescence of CAT standard serial dilutions subtracted from that of a no-catalase control.

*Protein content*

All enzyme activities were normalized to the total protein content, which was measured a commercially available kit. (#B6916, Bradford Assay Kit, Merck, Germany).

**Supplementary Table 1 -** Lethality data during the 21-day exposures

*A) Experiment 1*

| Time point | control | MP | UV-filter | MP+UV |
| --- | --- | --- | --- | --- |
|  |  |  |  |  |
| Living animals before treatment | 30 | 30 | 30 | 30 |
| **Living animals after treatment** | **30** | **29** | **25** | **29** |

*B) Experiment 2*

| Time point | control | MP | UV-filter | MP+UV |
| --- | --- | --- | --- | --- |
|  |  |  |  |  |
| Living animals before treatment | 60 | 60 | 60 | 60 |
| **Living animals after treatment** | **58** | **56** | **54** | **56** |

**References**

Acs A, Komaromy A, Kovacs AW, Fodor I, Somogyvari D, Gyori J, Farkas A (2023) Temperature related toxicity features of acute acetamiprid and thiacloprid exposure in *Daphnia magna* and implications on reproductive performance. Comp Biochem Physiol C Toxicol Pharmacol 268:109601. <https://doi.org/10.1016/j.cbpc.2023.109601>

Gaaied S, Oliveira M, Le Bihanic F, Cachot J, Banni M (2019) Gene expression patterns and related enzymatic activities of detoxification and oxidative stress systems in zebrafish larvae exposed to the 2,4-dichlorophenoxyacetic acid herbicide. Chemosphere 224:289-297. <https://doi.org/10.1016/j.chemosphere.2019.02.125>

Georgantzopoulou A, Cambier S, Serchi T, Kruszewski M, Balachandran YL, Grysan P, Audinot JN, Ziebel J, Guignard C, Gutleb AC et al. (2016) Inhibition of multixenobiotic resistance transporters (MXR) by silver nanoparticles and ions in vitro and in *Daphnia magna*. Sci Total Environ 569-570:681-689. <https://doi.org/10.1016/j.scitotenv.2016.06.157>

Le Bihanic F, Couillard CM, Rigaud C, Legare B (2013) A simple and reliable in vivo EROD activity measurement in single *Fundulus heteroclitus* embryo and larva. Mar Environ Res 84:17-23. <https://doi.org/10.1016/j.marenvres.2012.11.003>

Schiano Di Lombo M, Weeks-Santos S, Clerandeau C, Triffault-Bouchet G, Langlois Valerie S, Couture P, Cachot J (2021) Comparative developmental toxicity of conventional oils and diluted bitumen on early life stages of the rainbow trout (*Oncorhynchus mykiss*). Aquat Toxicol 239:105937. <https://doi.org/10.1016/j.aquatox.2021.105937>
